# Data supporting the validation of a simulation model for multi-component gas separation in polymeric membranes

**DOI:** 10.1016/j.dib.2016.10.019

**Published:** 2016-10-26

**Authors:** Lorena Giordano, Denis Roizard, Roda Bounaceur, Eric Favre

**Affiliations:** Laboratoire Réactions et Génie des Procédés (LRGP) (UMR 7274), Université de Lorraine, France

**Keywords:** Post-combustion CO_2_ capture, Membrane separation, Model validation, Separation performances

## Abstract

The article describes data concerning the separation performances of polymeric hollow-fiber membranes.

The data were obtained using a model for simulating gas separation, described in the research article entitled “Interplay of inlet temperature and humidity on energy penalty for CO_2_ post-combustion capture: rigorous analysis and simulation of a single stage gas permeation process” (L. Giordano, D. Roizard, R. Bounaceur, E. Favre, 2016) [1]. The data were used to validate the model by comparison with literature results. Considering a membrane system based on feed compression only, data from the model proposed and that from literature were compared with respect to the molar composition of permeate stream, the membrane area and specific energy requirement, varying the feed pressure and the CO_2_ separation degree.

**Specifications Table**

**Table:** 

Subject area	Process engineering
More specific subject area	Post-combustion CO_2_ capture
Type of data	Table and Figures
How data was acquired	Numerical simulation
Data format	Raw
Experimental factors	No pretreatment of data was performed
Experimental features	The simulation of single stage membrane system was performed using a proprietary software tool integrated in Aspen Plus environment
Data source location	Nancy, France
Data accessibility	Data are provided within this article

**Value of the data**

• The data describe the separation performances and the specific energy requirement of a single-stage membrane unit; hence they can be used in future works to compare simulation results of different membrane system models.
• The data contain key information regarding the performances of a single-stage membrane system operating the CO_2_ capture from exhaust flue gases; hence these data can be used to support the study of more complex membrane separation systems, such as those based on dual-stage configuration.
• The data can be used by other researchers for a preliminary assessment of energy penalty inflicted to power plants integrating a post-combustion CO_2_ capture system based on a single-stage membrane configuration.
• The data are valuable for other studies concerning the conceptual design of single-stage membrane systems for CO_2_ capture reuse applications.

## 1 Data

Data shared in this article concern the validation of a model for evaluating the performances of a post-combustion CO_2_ capture membrane system. Data consist of molar fractions of permeate stream, membrane area and specific energy requirement of membrane system. Data were obtained by varying the CO_2_ separation degree from 0 to 100% and the feed pressure from 4 bar to 10 bar.

## 2 Experimental design, materials and methods

**Fig. 1:**
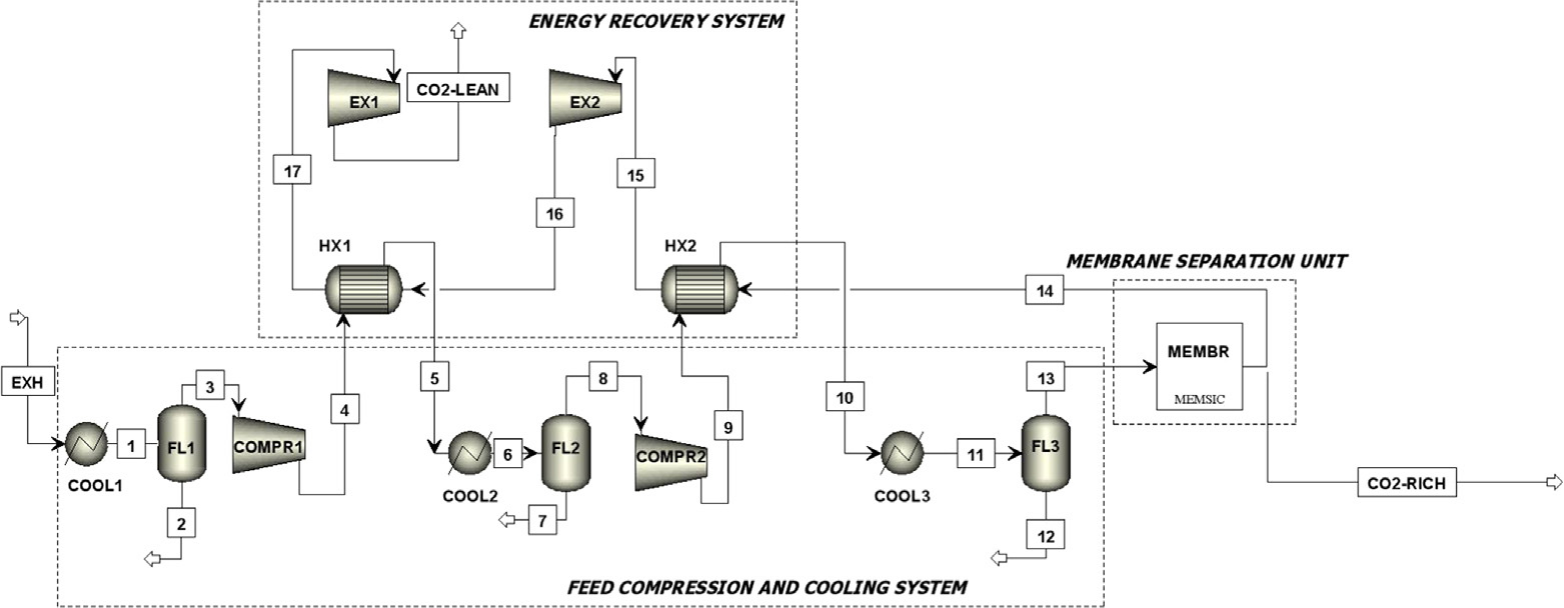
Layout of a single stage membrane system with feed compression only in Aspen Plus V8.4.

With the aim to validate a model for simulating the gas separation in polymeric hollow-fiber membrane modules [1], the related simulation data were compared with those obtained in a previous published paper by Low et al. [2], based on the same membrane system layout and operating conditions. Specifically, a single-stage configuration with feed compression only was simulated (Fig. 1). Gas separation in the membrane module was mimicked using the proprietary simulation tool M3PRO [3]; the latter was integrated in Aspen Plus environment [4], with the aim to simulate the energy behavior of the whole membrane separation system.

Table 1 summarizes the simulation operating conditions, including the membrane separation properties and the thermodynamic conditions of flue gas to be treated.

**Fig. 2:**
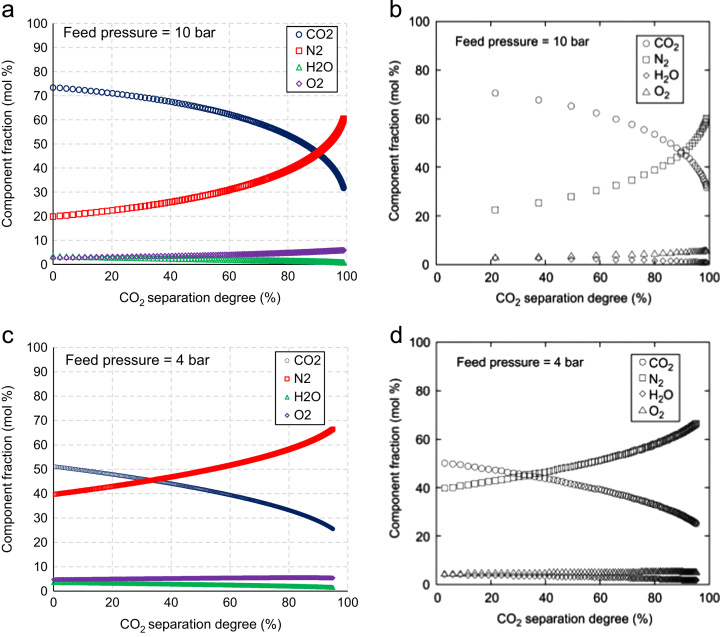
Comparison between the permeate molar composition evaluated with the model proposed (a and c) and that from Low et al. [2] (b and d).

Fig. 2 compares the permeate composition evaluated with the model proposed (Fig. 2a and c) and that obtained by Low et al. [2] (Fig. 2b and d), varying the CO_2_ separation degree and the feed pressure. It is noted that the trend of simulated data varying the CO_2_ separation degree fits very well with the literature data. For instance, setting a feed pressure of 10 bar and increasing the CO_2_ separation degree from 20% to 100%, Fig. 2a shows that in the proposed model CO_2_ molar fraction reduces from around 70% to 30% and N_2_ molar fraction increases from around 20% to 60%. Almost the same values are observed in Fig. 2b, depicting the trend of permeate molar fractions evaluated in [2] with the same operating conditions. Additionally, both models show that CO_2_ and N_2_ molar fractions attain the same values (≈45%) for a CO_2_ separation degree of around 90%. The good agreement is also confirmed at a lower feed pressure (4 bar), where both models show that CO_2_ and N_2_ molar fractions pass from 50% to 25% and from 40% to less than 70% respectively, for a corresponding increase of CO_2_ separation degree up to 95% (Fig. 2c and d).

**Fig. 3:**
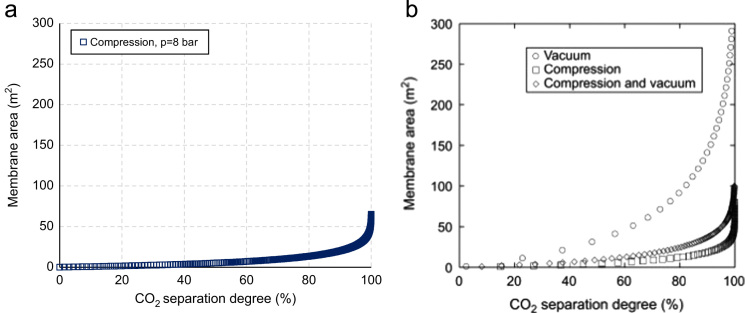
Comparison between membrane area evaluated with the model proposed (a) and that from Low et al. [2] (b) for a feed pressure of 8 bar.

Fig. 3 compares the membrane area evaluated with the model proposed, for a feed pressure of 8 bar (Fig. 3a) and that obtained by Low et al. [2] (Fig. 3b) at the same operating conditions. The trend of membrane area evaluated with the proposed model fit well with that obtained in [2]. In this regards, it is noted that for both models membrane area has an exponential increase, stating at around 20 m^2^ for a CO_2_ separation degree of 90%.

**Fig. 4:**
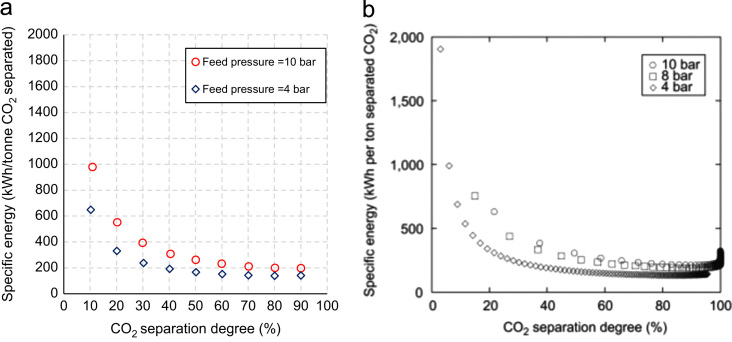
Comparison between specific energy evaluated with the model proposed (a) and that from Low et al. [2] (b) for a feed pressure of 4 bar and 10 bar.

Finally, Fig. 4 allows to compare the model proposed and that in [2] in terms of specific energy requirement for CO_2_ separation, assuming feed pressures values of 4 bar and 10 bar. Specific energy requirement exhibits an exponential decreasing trend in both models; values evaluated by the model proposed are comparable or slightly lower than that in [2], due to a slight difference in membrane system layout. Indeed, in the configuration proposed, the feed compression system is thermally integrated with the dual stage turboexpander. This aspect allows to concurrently reduce the power consumption for compression and increase the energy production from the retentate expansion, thus positively affecting the net power consumption and the specific energy requirement. As a result, setting a CO_2_ separation degree of 90%, specific energy requirement for a feed pressure of 4 bar states at less than 150 kWh/tonne CO_2_ separated in both models; increasing feed pressure to 10 bar, specific energy states at less than 250 kWh/tonne in [2], while it reduces to around 200 kWh/tonne in the proposed membrane system.

## Figures and Tables

**Table 1:** Operating conditions for simulating the single stage membrane system with feed side compression.

Exhaust flue gas		Membrane	
Parameter	Value	Parameter	Value
x_CO2_, % mol	14.7	p_CO2_, Barrer	150
x_N2_, % mol	76.2	p_N2_, Barrer	4.2
x_H2O_, % mol	4.3	p_H2O_, Barrer	1500
x_O2_, % mol	4	p_O2_, Barrer	11.7
x_Ar_, % mol	0.8	p_Ar_, Barrer	11.7
m_EXH_, Nm^3^/h	100		
T_EXH_, °C	30		
p_EXH_, bar	1		
